# Editorial: Spotlight on North America – chemical sciences

**DOI:** 10.3389/fchem.2024.1531189

**Published:** 2024-12-03

**Authors:** Subhash C. Sinha, Chinna Rajesh Ummadisetti, Kenneth K. Laali, Kai Bao, Naga V. S. D. K. Bhupathiraju, Iwao Ojima

**Affiliations:** ^1^ Helen and Robert Appel Alzheimer’s Disease Research Institute, Brain and Mind Institute, Weill Cornell Medicine, New York, NY, United States; ^2^ MilliporeSigma, Miamisburg, OH, United States; ^3^ Department of Chemistry, Hunter College, City University of New York, New York, NY, United States; ^4^ Department of Chemistry and Biochemistry, University of North Florida, Jacksonville, FL, United States; ^5^ Massachusetts General Hospital, Harvard Medical School, Boston, MA, United States; ^6^ Department of Chemistry and Institute of Chemical Biology and Drug Discovery, Stony Brook University, Stony Brook, NY, United States

**Keywords:** toxicology testing, nitration of aromatics, redox active ligand system, high-refractive index low absorbance adhesives and plastics, cheminformatics and artificial intelligence, reduction of β-amyloid production

This Research Topic of three reviews and four original articles by seven internationally recognized North American-based researchers presents a glimpse of the huge number of significant works in the area of Analytical, Inorganic, Organic, Medicinal and Pharmaceutical, Polymer, and Green and Sustainable Chemistry. The works presented here highlight advances in theory, experiment, and methodology with applications to solving compelling problems.

Development of an efficient and highly sensitive analytical tool for toxicology plays a critical role in clinical, forensic, and doping control applications. In the Analytical Chemistry Section, Kenichi Tamama (University of Pittsburgh) reported a Liquid Chromatography-Mass Spectrometry (LC-MS) based method for detecting drug contents present in urine samples, which are protein-poor liquid specimens (Tamama). This high resolution LC-MS analysis method can provide the most convincing results quickly in busy toxicology laboratories for drug screening. The pros and cons of the LC-MS method were critically discussed in this article.

Electrophilic aromatic nitration is a widely used process for producing a variety of dyes, agrochemicals, high energy materials, fine chemicals and pharmaceuticals. In the Green and Sustainable Chemistry Section, Béla Török and his coworkers (University of Massachusetts Boston) reported an Original Research Article describing environmentally benign methods for electrophilic aromatic nitrations (Plasse et al.). The method using 11–15.8 M aqueous nitric acid is certainly safer compared to using fuming nitric acid (90%, 21.4 M) and in some cases ultrasound helped to facilitate the reaction. Furthermore, these conditions avoid the double nitration of the substrates, which is a significant advantage over the conventional processes, using fuming nitric acid or a strong co-acid.

In the Inorganic Chemistry Section, David J. R. Brook (San Jose State University) and his collaborators reported their investigation into the electronic structure of zinc and nickel metal complexes with dipyridylverdazyl ligand using electrochemistry and spectroscopy techniques in an Original Research Article (Fleming et al.). The change in redox events on the ligand affects the type of interaction with metal *d*-orbitals, switching between a sigma/pi donor and pi acceptor. These results will provide a solid basis for the understanding of the other transition metal compounds with this ligand, as well as for the design of other valence tautomeric systems.

In the Polymer Chemistry Section, James M. Eagan and coworkers (The University of Akron) reported the metal-salen (M = Al, Cr, Co: Cr = metal of choice)-catalyzed copolymerizations of epoxides and CS_2_ for preparing high-refractive index (n up to 1.73) with low absorbance (κ < 0.005) adhesives and plastics in an Original Research Article (Schwarz et al.). Polymer synthesis using this approach is transformative for optoelectronics and advanced optics that require transparent high-performance materials. The use of metal-salen catalysts enables controlled polymerization, ensuring uniformity and desirable material characteristics, such as optical clarity and thermal stability.

In the Organic Chemistry Section, Yannick Djoumbou-Feunang (Corteva Agriscience, Indiana) and his collaborators, have provided an excellent overview of how artificial intelligence (AI)- and cheminformatics-driven molecule discovery could help finding new pesticides for safe use in agriculture in a Review Article (Djoumbou-Feunang et al.). AI and cheminformatics have made significant progress over the years and play critical roles in the discovery of bioactive molecules. Combining these ITs with the traditional technology could address the needs and challenges of agrochemical discovery towards rapidly developing novel and more sustainable products. The authors also recognize the challenges in the wider adaptation of the *in silico* approach, due to the scarcity of the standardized high-quality agrochemical datasets.

In the Medicinal and Pharmaceutical Chemistry Section, Jerry Yang and his coworkers (University of California San Diego) contributed a timely Review Article (Teppang et al.) on the recent development of fluorescent probes that can detect oligomeric aggregates of amyloidogenic proteins, including amyloid-β, tau and synuclein, present in Alzheimer’s and Parkinson’s disease patients. The authors highlighted the guidelines for designing potentially advanced fluorophores with improved specificity for oligomers and discussed fluorescent probes that bind various aggregation states of the proteins. Indeed, some advancements have been made toward probes to detect amyloid-β oligomers, while similar studies to identify probes to detect tau and synuclein oligomers have proven difficult.

Also, in the Medicinal and Pharmaceutical Chemistry Section, William J. Netzer and his coworkers (The Rockefeller University, New York) as well as Subhash C. Sinha (The Rockefeller University and Weill Cornell Medicine, New York) reported the synthesis and evaluation of novel regio-isomers of imatinib in cell models of Alzheimer’s disease in an Original Research Article (Netzer et al). The authors showed that several new analogs reduce β-amyloid production by modulating both β- and γ-secretase cleavages of amyloid precursor protein (APP) similarly to imatinib. These compounds provided examples of how the fragment hopping strategy can provide entirely new classes of compounds, yet they maintain comparable activities.

In summary, the diverse Research Topic of the articles not only presented novel findings in each field of chemistry, but also indicated new and promising directions to explore in the years to come.

